# The suppressive functions of Rora in B lineage cell proliferation and BCR/ABL1-induced B-ALL pathogenesis

**DOI:** 10.7150/ijbs.68939

**Published:** 2022-03-06

**Authors:** Ning Li, Nan Wang, Wei He, Yunyu Feng, Qiang Qiu, Huandi Qiu, Li Zheng, Yuexia Yin, Bochuan Wang, Yuanyuan Sun, Cong Pan, Klarke M. Sample, Juan Huang, Zhiguang Su, Zhihui Li, Haojian Zhang, Yiguo Hu

**Affiliations:** 1 Department of Thyroid Surgery and National Clinical Research Center for Geriatrics, State Key Laboratory of Biotherapy and Cancer Center, West China Hospital, Sichuan University, and Collaborative Innovation Center for Biotherapy, Chengdu, P.R. China.; 2 Laboratory of thyroid and parathyroid disease, Frontiers Science Center for Disease-related Molecular Network, West China Hospital, Sichuan University, Chengdu, China.; 3 Institute of Life Science, eBond Pharmaceutical Technology Ltd., Chengdu, China; 4 Sichuan Provincial People's Hospital, Chengdu, Sichuan; 5 Molecular Medicine Research Center and National Clinical Research Center for Geriatrics, West China Hospital, and State Key Laboratory of Biotherapy, Sichuan University, Chengdu 610041, China; 6 Department of Thyroid Surgery, West China Hospital of Sichuan University, Chengdu, China.; 7 The State Key Laboratory Breeding Base of Basic Science of Stomatology & Key Laboratory of Oral Biomedicine Ministry of Education, School & Hospital of Stomatology, Wuhan University, Wuhan, China; Frontier Science Center for Immunology and Metabolism, Medical Research Institute, School of Medicine, Wuhan University, Wuhan, China.

**Keywords:** *RORA*, B cell, proliferation, development, Philadelphia positive B-ALL

## Abstract

RORA plays an important role in regulating circadian rhythms, inflammation, metabolism and cellular development. Herein, we explore the roles of *Rora* in B cell proliferation and differentiation, as well as in Ph^+^ B-ALL. By using *Rora^loxp/loxp^* Mx-1-Cre mice, *Rora* was deleted in hematopoietic cells post Pipc induction. *Rora* deficiency mice were associated with an obvious accumulation of B cells in the peripheral blood, bone marrow, and spleen. On the other hand, activation of Rora with Cholesterol sulfate (CS) was associated with decreased B cell numbers. RNA-seq analysis revealed that the transcription level of Lmo1 was decreased in *Rora* deficient B cells. Moreover, the expression of *RORA* was shown to be decreased in Ph^+^ B-ALL cells compared to peripheral blood derived B cells from healthy donors. The overexpression of Rora in BaF3 cells with BCR/ABL1 was also associated with impeded the cell growth and an increased apoptotic rate compared to cells transduced with BCR/ABL1 alone. The co-expression of BCR/ABL1 and Rora induced B-ALL mouse model was associated with the significant inhibition of BCR/ABL1-transformed cell growth and prolonged the survival of the diseased mice. These results suggest a novel role for *Rora* in B cell development and Ph^+^ leukemogenesis.

## Introduction

RAR related orphan receptor A (RORA) is a widely expressed transcription factor that belongs to the nuclear hormone receptor superfamily. RORA can bind as either a monomer or a homodimer to upstream hormone response elements, leading to enhanced expression of specific target genes^1^. RORA is a key regulator in embryonic development, cell differentiation, immunity, circadian rhythms, and the metabolism of lipid, steroid, xenobiotic and glucose^1^. As such, RORA plays a major role in the development and cell differentiation of many tissues and is required for normal development of the cerebellum^2^. RORA is also involved in a variety of diseases, including muscle atrophy, immunodeficiency, osteoporosis and atherosclerosis^1^. In the vascular system, RORA has been shown to be associated with angiogenesis and the contractile function of smooth muscle cells following ischemia^3^.

RORA exerts its anti-inflammatory activity by inhibiting the NFKB signaling pathway. RORA has been shown to induce the transcription of IKΒA to restrict NFKΒ translocation to the nucleus, thereby preventing it from fulfilling its regulatory function and limiting the expression activation of its target genes, including cytokines^4^. RORA regulates granulosa cell proliferation and participates in calcium-mediated signaling through the regulation of *SHH* expression^2^. Moreover, RORA is involved in a number of intracellular signaling transductions and cell stress response events. For example, RORA binds to E2F1 to suppress its transcriptional activity in epithelial cells^5^. DNA damaging agents are also able to induce the (TP53-dependent) expression of *RORA*, since it is a direct target of TP53^6^. RORA also interacts with HIF1A and activates its transcriptional activity to participate in hypoxia signaling, suggesting that RORA participates in the control of gene transcription in response to hypoxic stress^7^.

The role of RORA as a tumor suppressor has been shown in a variety of tumors^6^,^8^-^10^. RORA is downregulated in breast tumors compared to healthy breast tissue, where the expression of RORA suppresses the malignant phenotype of breast cells^9^. The activation of RORA in prostate cancer cells has also been associated with decreased S phase cell numbers and a significant suppression of proliferation^10^. Previous reports have demonstrated that RORA functions as a transrepressor for WNT/B-catenin in colon cancer, at the crossroads between the canonical and the noncanonical WNT signaling pathways^8^. Moreover, RORA is known to function as an important negative modulator of angiogenesis in breast cancer to suppress its progression^11^.

However, the involvement of *RORA* in B cell development and differentiation, as well as the pathogenesis and progression of Philadelphia chromosome positive (Ph^+^) acute B cell leukemia (Ph^+^ B-ALL), has not been studied. Ph^+^ is a fusion chromosome, which is generated from the translocation of chromosome 9 and 22, t (9;22). The fusion results in generating an oncogene, *BCR-ABL1*, which contributes to about 20 % to 30 % of adults B-ALL^12^. Current treatment strategies, such as TKI and CAR-T therapies, have benefited most patients, but the prognosis for some patients is still poor. It has been shown that NFKB^13^,^14^, E2F1^15^, WNT/B-catenin^16^, as well as HIF1A^17^ signaling pathways play critical roles in the development and progression of Ph^+^ leukemias. Therefore, we formed the hypothesis that RORA was probably involved in B cell development and the progression of Ph^+^ B-ALL (particularly BCR/ABL1-induced B-ALL pathogenesis).

## Materials and Methods

### Mice

All the wild type (WT) mice were of the C57BL/6J (CD45.2) background purchased from the Jackson Laboratory (US) and were bred and maintained in the Animal Center of the State Key laboratory of Biotherapy at Sichuan University (CN). C57BL/6J *Rora^loxp/loxp^* conditional knockout mice were created by Biocytogen (CN) and Mx-1-Cre mice were obtained from the Jackson Laboratory. *Rora^loxp/loxp^*Mx-1-Cre mice were generated by breeding *Rora^loxp/loxp^* mice with Mx-1-Cre transgenic mice. Littermates with a *Rora^loxp/loxp^* genotype were used as controls and treated with the same conditions as *Rora^loxp/loxp^*Mx-1-Cre mice. Both male and female mice (8-12 weeks old) were used for the experiments, and age and gender-matched littermates were used as controls. All the animal studies were performed in accordance with the guidelines approved by the Animal Care and Use Committee of the State Key laboratory of Biotherapy, Sichuan University.

### Polyinosinic-polycytidylic acid injection to induce Rora deletion

Polyinosinic-polycytidylic (Pipc) acid sodium salt (Sigma-Aldrich, US) was dissolved in Phosphate-buffered saline (PBS) at a concentration of 1mg/ml and stored as a stock solution at -20°C. Eight weeks (wks) post birth *Rora^loxp/loxp^*Mx-1-Cre and *Rora^loxp/loxp^* (WT) mice were administered an intraperitoneal (IP) Pipc injection (15 mg/kg body weight) once every two days with a total of five doses, which enabled the generation of mice with a specific* Rora* deletion in the blood system. The mice were sacrificed four, eight and 12 (wks) post Pipc treatment, and cells from peripheral blood (PB), spleens (SPL) and bone marrow (BM) cells in femurs and tibia were harvested.

### Histopathology and Immunohistochemistry

SPL and BM were fixed, processed, sectioned and stained with hematoxylin-eosin (H&E) using a standard protocol. Femur bones were treated for an additional hour in decalcifying solution (Thermo Fisher Scientific, CN). The immunohistochemical analysis was performed according to standard protocol using a rat anti-mouse B220 (RA3-6B2) monoclonal antibody (Santa Cruz Biotechnology, US).

### FACS analysis of B cells

All flow cytometric analyses and cell sorting were performed on a BD FortessaTM X or BD Fluorescence-activated cell sorting analysis (FACS) AriaTM III and analyzed using FlowJo V10 software at the Core Facility in the State Key Laboratory of Biotherapy at Sichuan University. BM, SPL, PB cells were stained according to standard procedures, and samples were analyzed on a BD FortessaTM X flow cytometer. Single cell suspensions were incubated for 15 minutes at room temperature with various combinations of the following cell-surface marker antibodies (Thermo Fisher Scientific, US): B220, B220^+^IgM^-^, B220^low^IgM^+^ and B220^high^IgM^+^ for B cells, pro/pre-B cells, immature B cells and mature B cells, respectively.

### Cell Immunofluorescence Staining and Imagining

Freshly sorted BM cells were fixed with BD Cytofix Fixation Buffer (BD Biosciences) at room temperature for 30 minutes and seeded onto fibronectin-coated glass slides in Hank's Balanced Salt Solution with 10% FBS. Prior to the immunostaining, the cells were washed with 1x Phosphate-Buffered Saline (PBS) and permeabilized with 0.2% Triton X-100 in PBS for 20 minutes and blocked with 10% Donkey Serum (Sigma-Aldrich, US) for 30 minutes. The immunostaining was performed using a primary anti-Rora antibody (Santa Cruz Biotechnology, US) with a secondary anti-goat AMCA-conjugated antibody, or a primary anti-Clock (Santa Cruz Biotechnology, US) with a secondary anti-rabbit DyLight549-conjugated antibody. Coverslips were mounted using ProLong Gold Antifade Reagent with DAPI (Thermo Fisher Scientific, CN).

### B-ALL mouse model

Femur and tibia BM cells were harvested from *Rora^loxp/loxp^* donor mice and their WT littermate. The BM cells were cosedimented with MSCV-BCR/ABL1-IRES-eGFP-IRES-iCre retroviral stock at 1000g for 90 minutes at 37ºC. C57BL/6J donor BM cells were simultaneously cosedimented with MSCV-BCR/ABL1-IRES-eGFP or MSCV-BCR/ABL1-IRES-eGFP-IRES-Rora retroviral stock at 1000g for 90 minutes at 37ºC. The transduced BM cells (1x10^6 cells/mouse) were subsequently transplanted via tail vein injection into recipients with the same genetic background, which received two doses of 5Gy of X-ray irradiation separated by 3 hours before BM cell transplantation. Leukemogenesis (GFP^+^B220^+^ biomarker) was analyzed using FACS in the recipients at multiple time points post transplantation.

### RNA Sequencing Analysis

Pro-B cells (B220^+^CD43^+^) were isolated from the BM of *Rora^loxp/loxp^*Mx-1-Cre and *Rora^loxp/loxp^* mice at week four post Pipc injection with a fluorescence activated cell sorter Aria III (Becton Dickinson). Total RNA was extracted for RNA sequencing with Trizol reagent (Thermo Fisher Scientific, US). Poly (A) mRNA was subsequently purified from 1 mg total RNA using the NEBNext Poly (A) mRNA Magnetic Isolation Module. A NEBNext Ultra Directional RNA Library Prep Kit (NEB, US) was used for the library preparation. Subsequently, the cDNA libraries from each individual mouse were sequenced on the Illumina MiSeq system to obtain paired-end reads (length = 150 bases). Data were analyzed with standard procedure and original data were provided in supplemental table S1 (BM pro-B cell) and S2 (B-ALL B cell).

### Statistical analysis

The data was presented using the mean ±SEM were appropriate. Statistical analyses were performed using the Mann Whitney U test using PRISM software (GraphPad 6 Software) to compare the differences between the treatment and control groups assuming equal variance. P=<0.05 was used as the threshold for statistical significance. *, **, *** and **** indicates P=<0.05, P=<0.01, P=<0.001 and P=<0.0001, respectively; whereas NS indicates not significant.

## Result

### *Rorα* deficiency increases the number of pan B lineage cells

Targeted ablation of *Rora* in mouse hematopoietic cells to determine whether Rora is involved in the regulation of B cell development. CRISPR/Cas9 technology was used to construct a *Rora^loxp/loxp^* conditional knockout mouse strain (Figure S1-1), these mice when bred to produce *Rora^loxp/loxp^*Mx-1-Cre mice enable the induced ablation of *Rora* via a Pipc tail injection. *Rora^loxp/loxp^* littermates were used as controls and treated with the same conditions for the experiments contained herein. To observe the overall effect of *Rora* deletion on peripheral leukocytes the ratio status of major cell populations was analyzed at weeks four, eight, and 12 post Pipc induction. Firstly, the deletion of *Rora* in cells isolated from the peripheral blood of these mice were examined using immunofluorescence staining with a Rora antibody and found that Rora protein level was decreased post Pipc treatment compared to control group cells (Figure S1-2 A).

**Figure 1:**
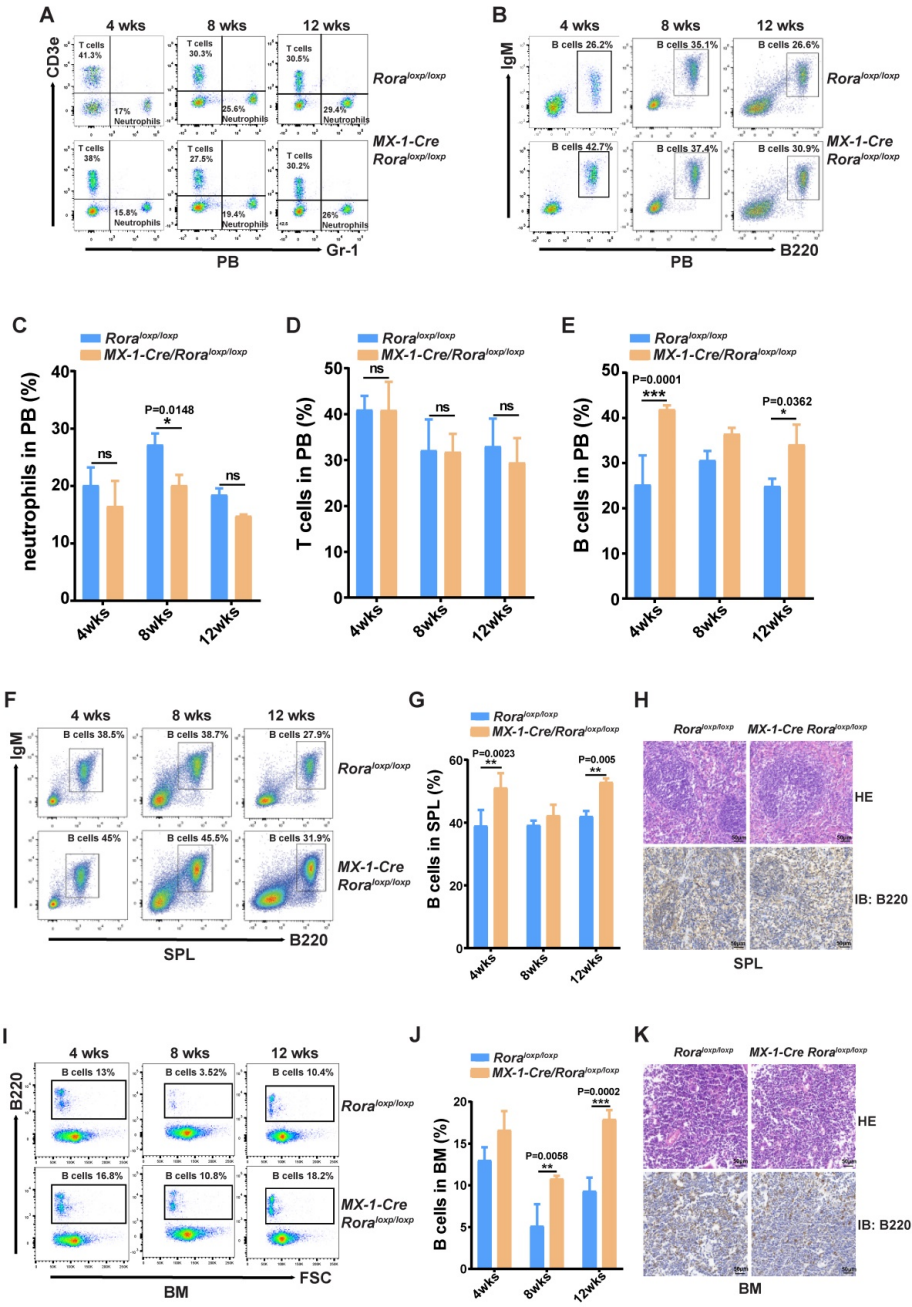
***Rora* deficiency increases B cell proliferation.** (**A, B, C, D** and** E**) A FACSanalysis detailing the percentage of neutrophils, T cells and B cells in PB from *Rora^loxp/loxp^* and Mx-1-Cre/*Rora^loxp/loxp^* mice at four, eight, and 12 weeks post Pipc treatment. (**F** and **G**) The FACS analysis and the percentage of mature B cell (B220^+^IgM^+^) in SPLs from WT mice and those with Rora deletion at four, eight, and 12 weeks post Pipc injection. (**H**) SPL sections for *Rora^loxp/loxp^* and Mx-1-Cre/*Rora^loxp/loxp^* mice treated with Pipc for eight weeks, stained with hematoxylin and eosin and B220. (**I** and** J**) The FACS analysis and the percentage of mature B cell (B220^+^IgM^+^) in the BM of WT and *Rora* depleted mice at four, eight, and 12 weeks post Pipc injection. (**K**) The SPL sections from *Rora^loxp/loxp^* and Mx-1-Cre/*Rora^loxp/loxp^* mice treated with Pipc for eight weeks were stained with hematoxylin and eosin and B220. All scale bars represent a size of 50μm. Where applicable the data was derived from three mice and each independent experiment was repeated three times (total of n=9). All values are stated using the mean ±SEM where applicable. The Mann Whitney U test was used to evaluate the significance, which was indicated using * (P=<0.05), ** (P=<0.01), *** (P=<0.001); ns (not significant).

**Figure 2:**
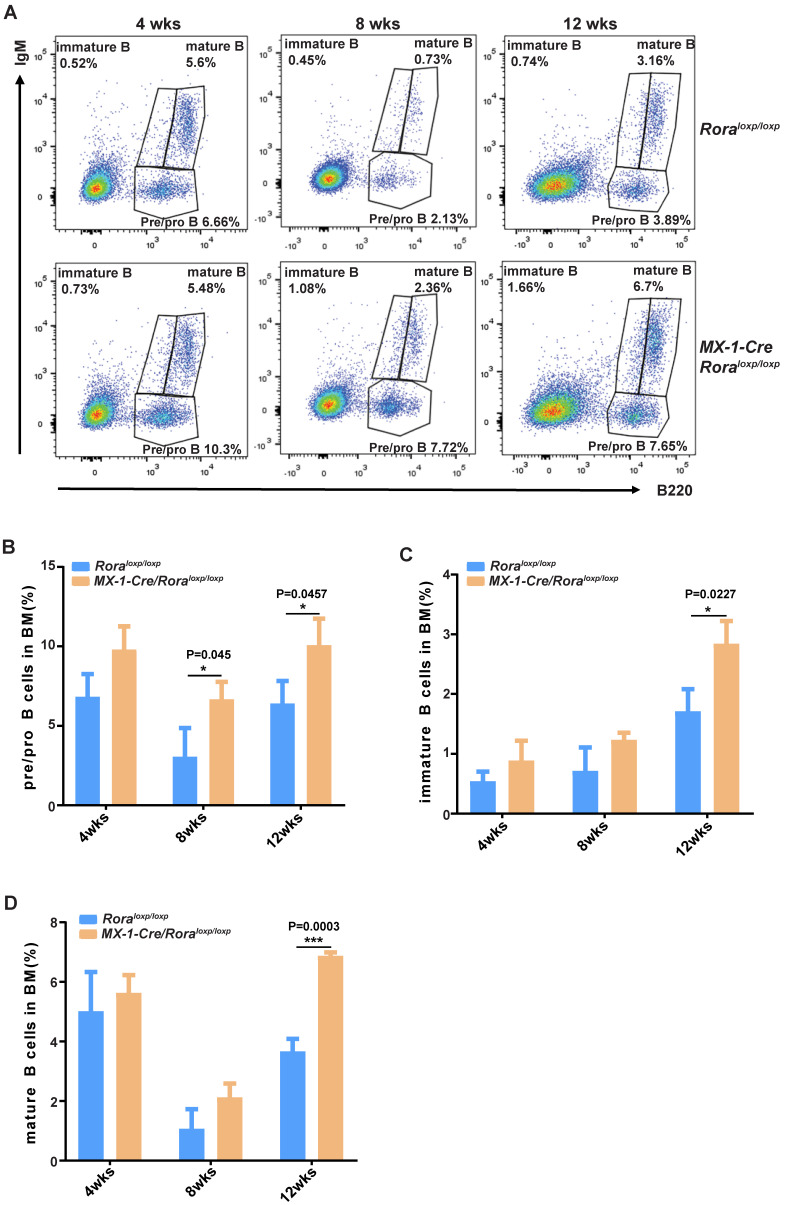
** The effect of *Rorα* deficiency on mature B cells in SPL and PBs**. (**A**) FACS analysis showing B-cell development stages (pro/pre-B cell represented with B220^+^IgM^-^, immature B cell represented with B220^low^IgM^+^; mature B cell represented with B220^high^IgM^+^) in BM at four, eight, and 12 weeks post Pipc treatment. (**B, C,** and** D**) The percentages of pro/pre-, immature, and mature B were derived from three mice and each independent experiment was repeated three times (total of n=9). All values are stated using the mean ± SEM where applicable. The Mann Whitney U test was used to evaluate the significance, which was indicated using * (P=<0.05), ** (P=<0.01), *** (P=<0.001); ns (not significant).

This enabled the observation that the percentages of neutrophils (Gr-1^+^) were significantly decreased (Figure 1A and C); B cell (B220^+^) percentages were increased (Figure 1B and E), and there were no obvious changes to T cells (Cd3^+^) (Figure 1A and D). These results indicated that B cells were more sensitive to *Rora* loss, therefore the percentage of B cells in the SPL and BM were also analyzed and found to be significantly higher in mice with a *Rora* deficiency (Figure 1F, G, I, and J), same to the absolute B cell numbers as well (Figure 1S-2 B and C). The B220 immunohistochemistry results were consistent with the FACS analysis, and showed that the distribution density of B-cells was increased* Rora* deficient mouse SPL and BM sections when compared to the controls (Figure 1H and K). These results demonstrate that *Rora* deficiency increases pan B lineage cells in BM and SPL.

FACS was used to delineate the role of Rora on B cell development in BM derived B cells with the following biomarkers: B220^+^IgM^-^, B220^low^IgM^+^ and B220^high^IgM^+^ for pro/pre-B cells, immature B cells and mature B cells, respectively (Figure 2A). The majority of B cell lineage populations in the BM were significantly increased in *Rora* deficient mice (Figure 2B, C, and D). These results indicate that Rora plays an important role in B development and that *Rora* deficiency causes increased numbers of B cell progenitors and maturation at all developmental stages.

### Gene expression profiling in *Rora* deficient B cells

**Figure 3:**
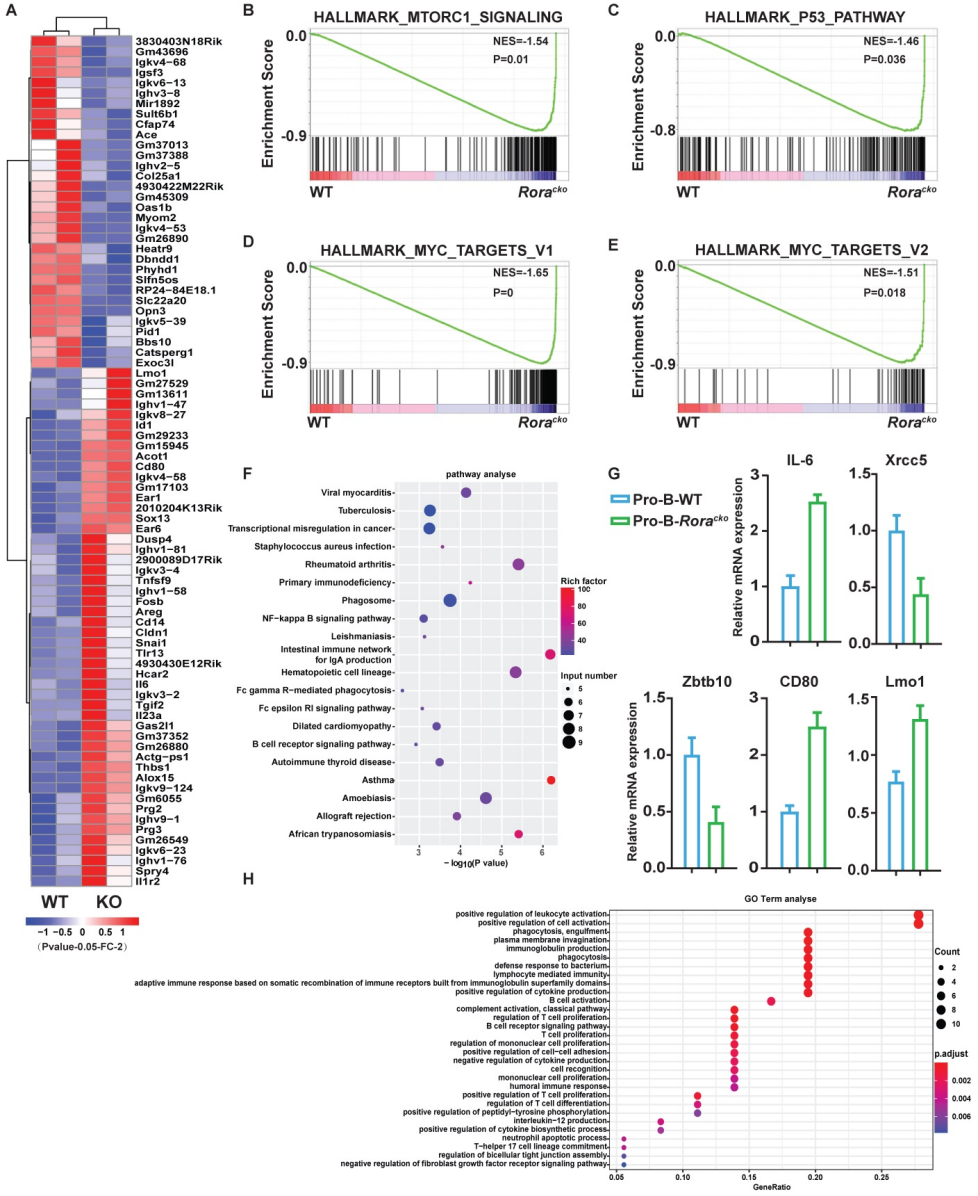
** Gene expression signatures of *Rora* deficiency pro-B cells.** (**A**) Heat maps showing the expression changes of top 82 genes andhierarchical clustering of the genes in *Rora* deficiency pro-B cells from the two biological replicates. Comparison of the global gene transcription profiles of *Rora* deficiency pro-B cells at week four after Pipc injection. (**B**-**E**) Gene Set Enrichment Analysis showsen richment of gene sets upregulated in *Rora* deficiency pro-B cells. (**F**) KEGG signal pathway enrichment analysis for the differentially expressed genes (DEGs) of pro-B cells from *Rora^loxp/loxp^* and Mx-1-Cre/*Rora^loxp/loxp^* mice. (**G**) Expression level of genes from selected gene sets were confirmed usingquantitative qPCR. Data are expressed as the means ± SD of triplicate experiments performed at one time. (**H**) A biological function-based GO term (BP) enrichment analysis for DEGs of pro-B cells from *Rora^loxp/loxp^* and Mx-1-Cre/*Rora^loxp/loxp^* mice.

To gain further insights into the roles of *Rora* in B cell development, two independent sets of pro-B cells were isolated from both groups of *Rora^loxp/loxp^Mx-1-Cre* and *Rora^loxp/loxp^* (WT) BM, which were treated with Pipc for 2 weeks. Total RNA was individually extracted from each sample, reversely transcribed to cDNA, and amplified. RNA sequencing was performed with the Illumina MiSeq system. Genes were considered significantly altered based on 1.5 fold or greater change in mean expression (P <=0.05). With these criteria, the expression of 405 genes was significantly decreased and 275 genes was increased. The top genes with obvious changes were listed in heatmap (Figure 3A). Among these genes, it is worth to highlight gene Lmo1, which mRNA level was significantly increased in *Rora* deficient pro-B cells. Previous studies have demonstrated that LMO1 is associated with ALL and other malignant progression, and LMO1 is a strong candidate for precursor B-cell leukemogenesis^18^-^20^.

To confirm the RNA-seq results, five genes (Il6, Xrcc5, Zbtb10, Cd80, and Lmo1), which were considered playing important roles in B cell development and functions, were selected and their mRNA levels were confirmed with qPCR. The qPCR results were consistent with RNA-Seq data (Figure 3G).

To further interpret the gene expression data, Gene Set Enrichment Analysis (GSEA) was performed. We found that genes involved in “mTORC1_signaling”, “MYC_targets”, and “P53_pathway” were increased and enriched in *Rora* deficient pro-B cells (Figure 3B-E). The results might explain the intracellular mechanism of *Rora* deficiency resulting in B cell high proliferation. The Differentially Expressed Genes (DEGs) were further analyzed for enrichment within KEGG pathways and Gene Ontology (GO) biological process (BP) terms. In the KEGG pathway analysis, the DEGs were significantly enriched in a number of pathways, including infection and cancer (Figure 3F). The GO BP analysis revealed that the DEGs were also significantly enriched in terms including cell activation and proliferation (Figure 3H). These results explained the cause of B-cell proliferation due to *Rora* deficiency at the molecular level.

### Pharmacologically activating Rora diminishes B cell numbers *in vivo*

**Figure 4:**
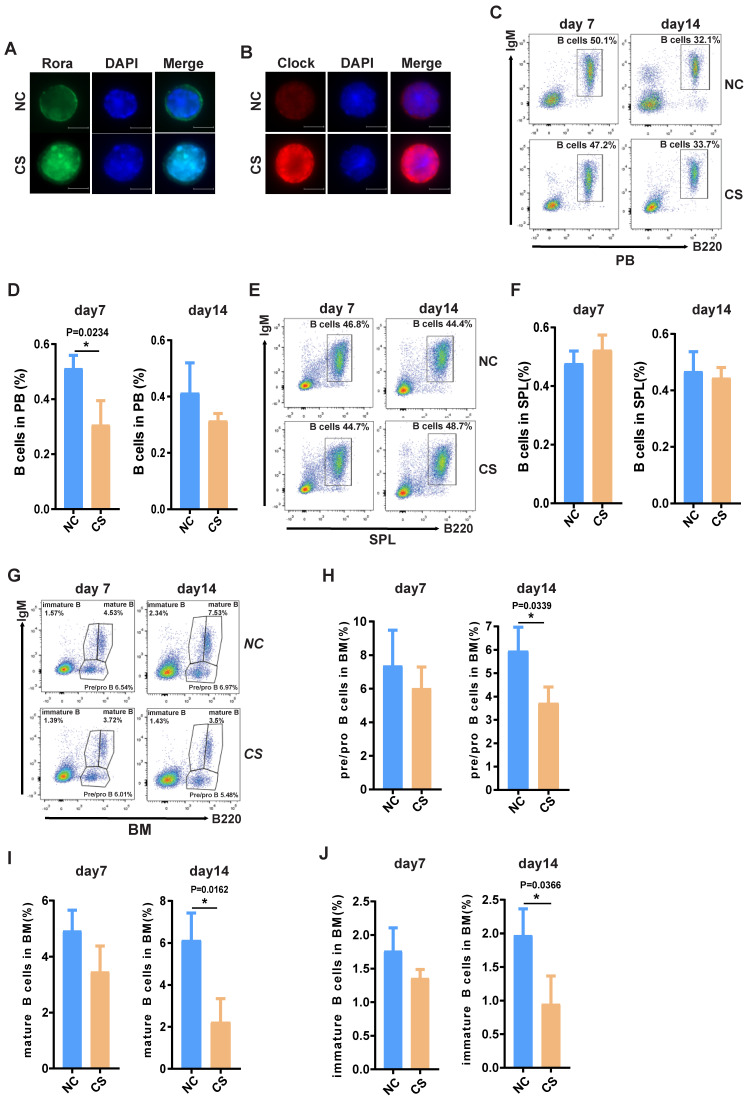
** The activation of Rora with CS inhibits B cell proliferation and differentiation.** (**A** ) Immunofluorescence (IF) staining of Rora (green), 4′,6-diamidino-2-phenylindole (DAPI), and merged in mouse BM cells isolated from the BM of 10-week old mice treated with CS for seven days. (**B**) IF staining of Clock (red), DAPI, and merged in mouse BM cells isolated from the BM of 10-week old mice treated with CS for seven days. (**C** and** D**) A FACS analysis showing the percentage and total number of mature B cells (B220^high^IgM^+^) in the PB of 10-week old mice treated with CS for seven and 14 days. (**E** and** F**) A FACS analysis of matures B cell (B220^+^IgM^+^) in the SPL and PB of 10-week old mice. (**G**) A FACS analysis demonstrating B-cell development stages (pro-and pre-B cell represented with B220^+^IgM^-^, immature B cell represented with B220^low^IgM^+^; mature B cell represented with B220^high^IgM^+^) in the BM. (**H, I,** and** J**). The percentage of pro/pre-, immature, and mature B cells were summarized and represented with bar charts with errorbars. Each independent experiment was repeated three times. All values were represented using the mean ±SEM, for which the Mann Whitney U test was used to evaluate the significance with a threshold of P=<0.05 (*).

To explore the possibility that increased Rora activity could impede B cell proliferation and development, wild type mice were treated with a specific Rora agonist, cholesterol sulfate (CS). Previous studies have reported that CS significantly increases Rora protein level and promoted Rora protein nuclear aggregation in cells^21^. Female wild type mice (10-weeks-old) were treated with 25mg/kg CS or PBS via IP injection and analyzed on days seven and 14 post treatment to determine the effects on B cell proliferation and development. Firstly, the level of Rora in cells isolated from the BM of the aforementioned mice were examined using immunofluorescence staining with a Rora antibody and found that Rora was significantly increased, as well as was aggregated in nuclear compared to control group cells (Figure 4A). To further confirm the biological activity of activated Rora, the function of *Clock* (its specific target gene) was also examined with immunofluorescence staining and found to be elevated in the CS treatment group (Figure 4B).

Furthermore, a PB B cell status analysis revealed that the total B cell (B220^+^) percentages were significantly decreased post CS treatment and that the decrease was persistent (Figure 4C and D). The percentage of splenic and BM associated B cells was also significantly decreased (Figure 4D, E, F, and G), as well the numbers (Figure S4A, B and C). The status of B cells differentiation was also analyzed using FACS with specific development biomarker antibodies. Post CS treatment there was a significant decrease for B cells with three different lineage markers that are critical development stages, namely: pro/pre-, immature, and mature B cells (Figure 2H and I). Cumulatively, these results indicate that increased Rora activity impedes B cell proliferation and development/differentiation.

### RORA involves in Ph^+^ B-ALL

**Figure 5:**
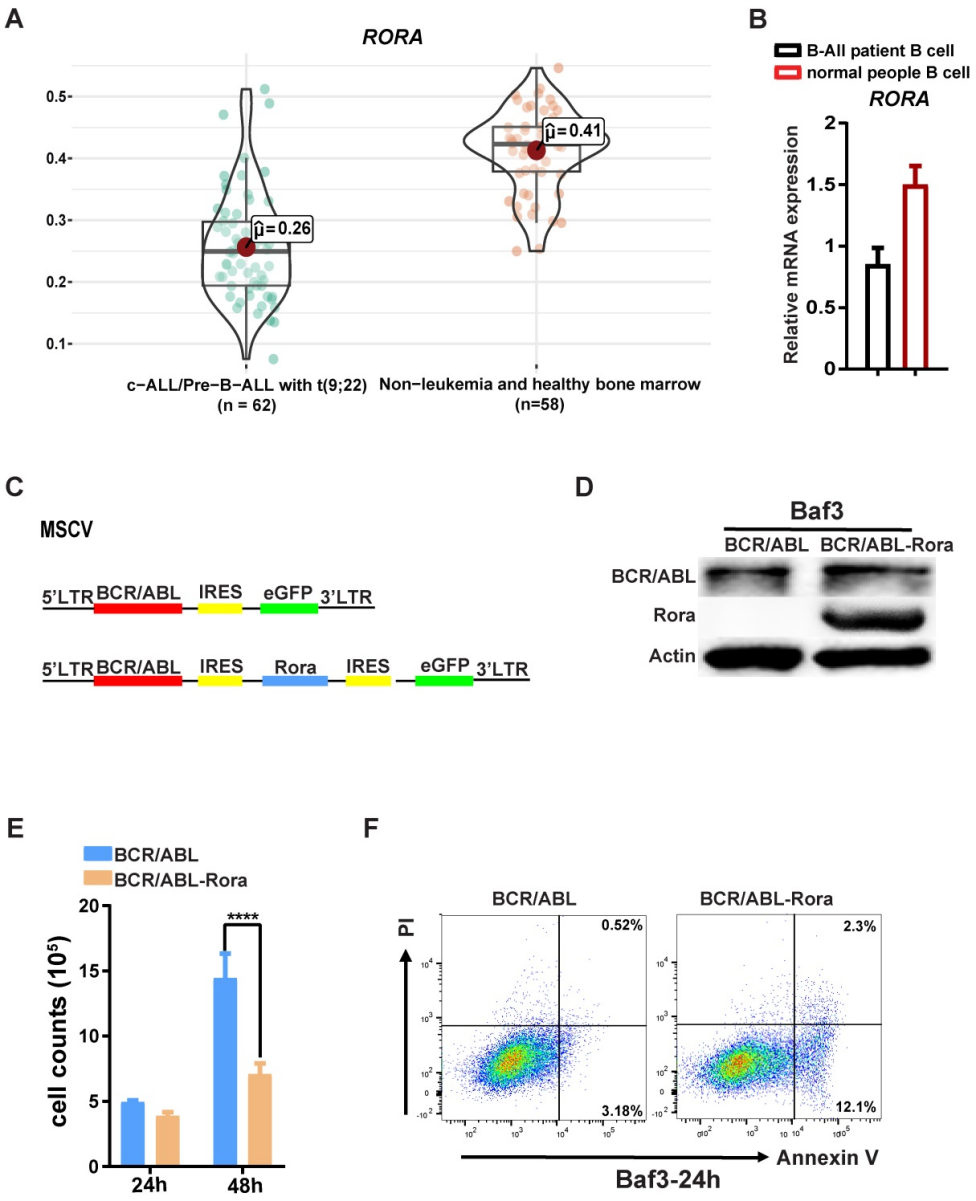
** RORA is involved in Ph+ B-ALL.** (**A**) A bioinformatics analysis showing the *RORA* expression status in Ph^+^ C-ALL/B-ALL and non-leukemia (n=62) and heathy BM (n=50). (**B**) The expression of *RORA* in human Ph^+^ B-All patient B cells (n=3) and healthy donor B cells were measured using real time-PCR (n=3). (**C**) A schematic diagram of MSCV-based retroviral vectors: BCR/ABL1 or BCR/ABL1-Rora. (**D**) A western blot analysis showing the expression of BCR/ABL1 and Rora in transfected BaF3 cells. Protein levels were determined for ABL1 (upper) and RORA (middle), with ATCB (lower) serving as the loading control. (**E**) The number of BCR/ABL1 or BCR/ABL1-Rora transformed BaF3 cells after 24 and 48 hours. (**F**) A FACS analysis to monitor the rate of apoptosis for BCR/ABL1 or BCR/ABL1-Rora-transduced BaF3 cells, the initial cell number is 1x10^6 cells/2ml. Each independent experiment was performed three times. Where applicable the values were represented using the mean ±SEM, for which the Mann Whitney U test was used to evaluate the significance with a threshold of P=<0.05 (*), *** indicates P=<0.001.

Due to the involvement of Rora in B cell proliferation and differentiation, further studies were conducted to examine whether RORA plays a role in Ph^+^ B-ALL. Firstly, an analysis of the dataset GSE13164 from GEO was conducted to determine the expression status of RORA in Ph^+^ B-ALL patient samples^22^, which revealed that *RORA* expression was decreased in leukemia cells compared to healthy PB derived B cells (Figure 5A). Confirmation of this analysis was conducted by qPCR (for *RORA* mRNA) in freshly isolated Ph^+^ B-ALL patient leukemia cells (B220^+^) and healthy donor B cells (B220^+^). The qPCR results were consistent with the dataset analysis, which revealed that RORA expression was significantly decreased in leukemia cells when compared to healthy B cells (Figure 5B).

To further elucidate the role of RORA in leukemia, transformed BaF3 cells were constructed using a retroviral vector to co-express RORA and BCR/ABL1 (Figure 5C) The successful generation of BaF3-BCR/ABL1-Rora and BaF3-BCR/ABL1 cells was confirmed using western-blotting with antibodies against ABL1 and RORA, both of which were highly expressed (Figure 5D). The higher expression of RORA affected the biological characteristics of the BCR/ABL1-transformed BaF3 cells, which had a lower proliferation rate (Figure 5E) and an increased rate of apoptosis (Figure 5F) when compared to BaF3-BCR/ABL1 cells. These results indicate that *RORA* has an expression pattern in Ph^+^ B-ALL that is akin to that of a tumor suppressor, which is matched by its effects on cell growth and apoptosis in BCR/ABL1 transformed BaF3 cells.

### *Rorα* deficiency accelerates BCR/ABL1-induced B-ALL progression in mouse

**Figure 6:**
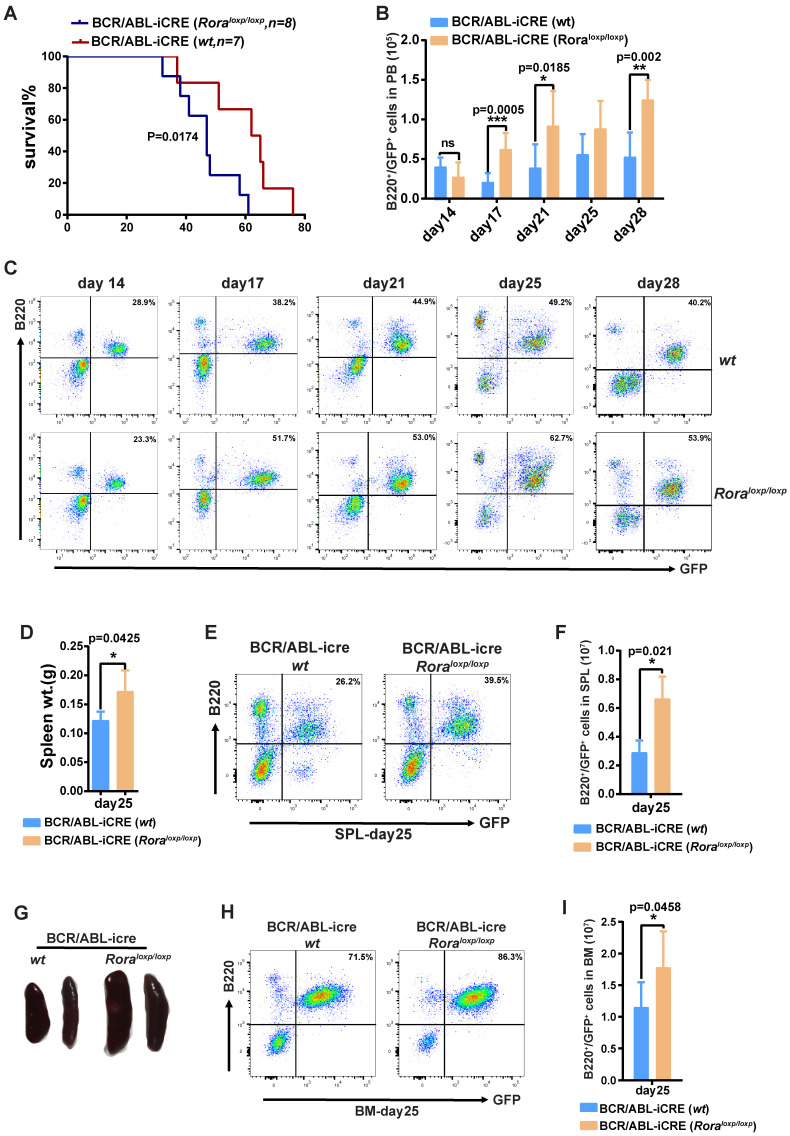
***Rorα* deletion accelerated BCR/ABL1-induced B-All progression in mouse.** (**A**) Kaplan-Meier B-All survival curves for hosts receiving BCR/ABL1-iCre-eGFP transformed BM cells from WT or* Rora^loxp/loxp^* mice. The number of recipients for each group and the survival days are indicated. (**B** and** C**) Percentage and total number of GFP^+^B220^+^cells in the PB of host mice receiving BCR/ABL1- iCre-transduced WT or *Rora^loxp/loxp^* donor BM cells at the indicated time points post BMT (n=5). (**D**) The gross appearance of the SPLs for host mice receiving BCR/ABL1-iCre-transduced WT or *Rora^loxp/loxp^* cells on day 25 post BMT. (**E**) The SPL weight for hosts receiving BCR/ABL1-iCre-transduced WT or *Rora^loxp/loxp^* cells on day 25 post BMT (n=3). (**F, G, H** and** I**). The percentage and total number of GFP^+^B220^+^cells in the SPLs and BM of host mice (n=3) receiving BCR/ABL1 or BCR/ABL1-Rora-transduced wild type mouse BM cells at the indicated time. Percentage and total number of leukemia cells are indicated where applicable. Each independent experiment was performed twice. All values are represented using the mean ±SEM, and the Mann Whitney U test was used to evaluate significance, which was indicated using * (P=<0.05), ** (P=<0.01), *** (P=<0.001); ns (not significant).

To further demonstrate the role of Rora on BCR/ABL1-induced B-ALL pathogenesis, a BCR/ABL1-induced B-ALL mouse model was developed. The model utilizes BM from *Rora^loxp/loxp^* and WT donor mice transduced with a retroviral vector containing BCR/ABL1 and iCre elements (to enable the ablation of endogenous *Rora*), which is transplanted into lethally irradiated recipient mice. Whilst all the recipients in both groups died due to the BRC/ABL1 induced B-ALL, the mice transplanted with *Rora^loxp/loxp^* BM died significantly faster (p=0.0174), with a median survival time of 46 days post BMT (BM transplant) verses 62 days for the WT group (Figure 6A). During disease progression, PB leukemia cells (GFP^+^B220^+^) were monitored on days 14, 17, 21, 25 and 28 post BMT, which revealed a significantly higher leukemia cell total and percentage in the *Rora* deficiency group recipients (Figure 6B and C). On day 25, three individual recipients from each group were sacrificed to enable a more detailed analysis of the leukemia progression. The mice in the group with *Rora* deletion had bigger and heavier SPLs (Figure 6D and E), which also contained a higher percentage and total number of leukemia cells (Figure 6D and E), similar results were observed for the BM (Figure 6H and I). These results indicated that *Rora* deficiency promoted BCR/ABL1-induced B-ALL progression.

To further reveal the molecular profiling changes in leukemia cells with *Rora* deficiency, BCR/ABL1 transformed pro-B cells were isolated from *Rora^loxp/loxp^* or WT donor groups and total RNA was extracted for RNA-seq. A similar analysis to normal pro-B cell RNA-seq data was performed as aforementioned. Totally, 269 genes were significantly decreased and 339 genes were increased. The top genes with obvious changes were listed in heatmap (Figure S6A). To confirm the RNA-seq results, seven genes were selected and the mRNA levels were confirmed with qPCR. The qPCR results were consistent with RNA-Seq data (Figure S6F).

GSEA results revealed that genes involved in “E2F_targets”, “G2M_checkpoint”, and “MYC_targets” were increased and enriched in *Rora* deficient leukemia cells (Figure S6B-E). These results well explained the reason *Rora* deficient promoted BCR/ABL1 transduced leukemogenesis and progress.

### Higher *Rora* expression impedes BCR/ABL1-induced B-ALL progression

**Figure 7:**
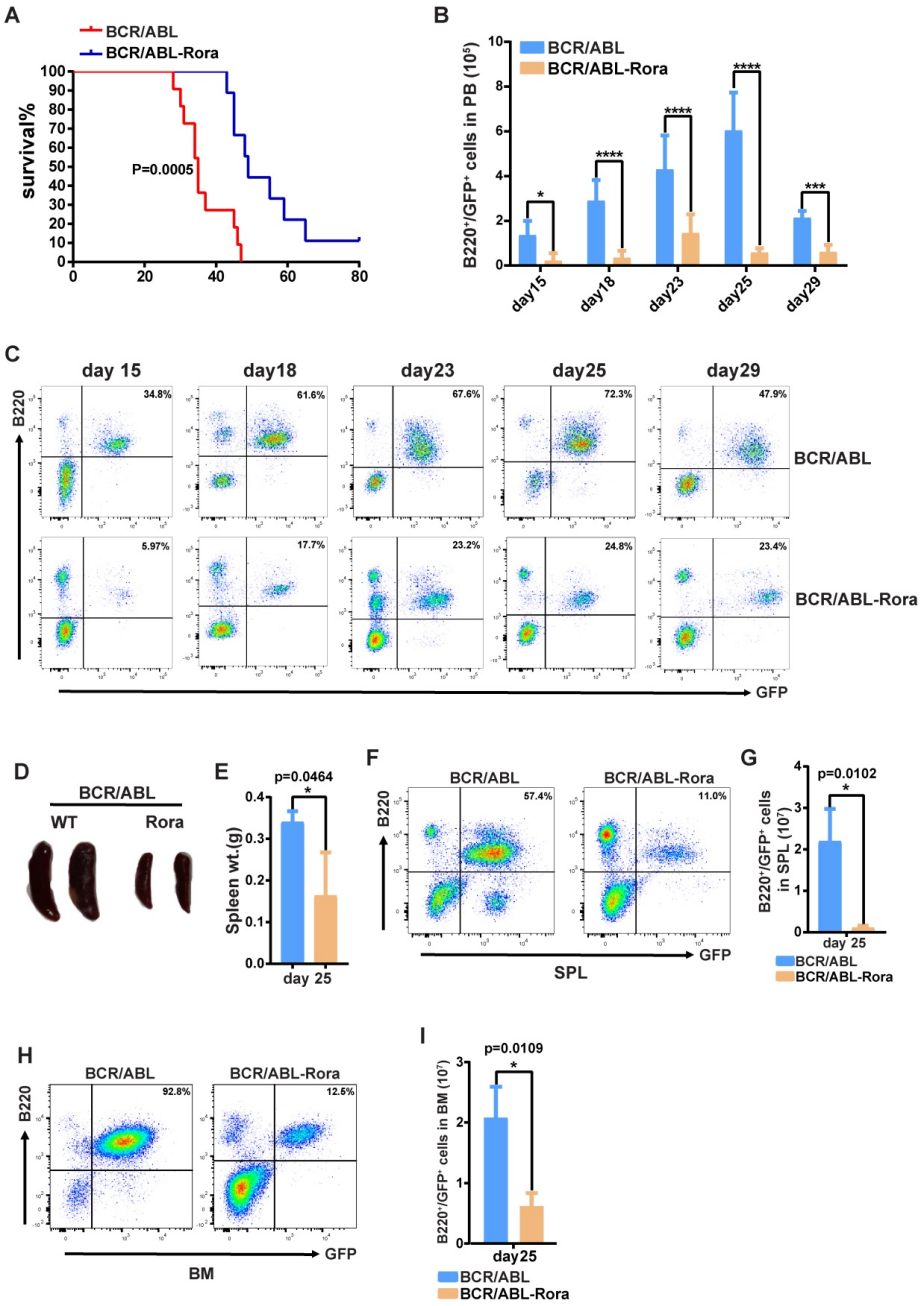
** High expression of Rora prevented BCR/ABL1-transduced B-ALL procession *in vivo*.** (**A**) Kaplan-Meier survival curves for recipients of BCR/ABL1 (n=9) or BCR/ABL1-Rora- (n=10) transduced wild type mouse BM cells. (**B**) The total numbers of GFP^+^B220^+^cells in PB of BCR/ABL1- or BCR/ABL1-Rora-transduced BM cells(n=5). (**C**) A FACS analysis showing the percentage of GFP^+^B220^+^cells in the PB from recipients of BCR/ABL1 or BCR/ABL1-Rora-transduced BM cells (n=5) at the indicated time point post BMT. (**D**) The gross appearance of the SPLs revealed splenomegaly in recipients of BCR/ABL1 or BCR/ABL1-Rora-transduced BM cells from WT donor mice 25 days after BMT. (**E**) The SPL weight hosts receiving BCR/ABL1 or BCR/ABL1-Rora-transduced BM cells (n=3). (**F, G, H** and** I**) The percentage and total number of GFP^+^B220^+^cells in the SPLs and BM of host mice (n=3) receiving BCR/ABL1 or BCR/ABL1-Rora-transduced wild type mouse BM cells the indicated times. Independent experiments were performed twice. The percentage and total number of leukemia cells are indicated, and represented using the mean ±SEM. The Mann Whitney U test was used to evaluates were considered significance with *P=<0.05, **P=<0.01, ***P=<0.001., which was indicated using * (P=<0.05), ** (P=<0.01), *** (P=<0.001); ns (not significant).

To investigate the effects of high *RORA* expression on leukemogenesis, wild type donor BM cells were transduced with virus containing BCR/ABL1 or BCR/ABL1-Rora, and transplanted into lethally irradiated recipient mice to induce B-ALL. To avoid transduction efficient differences between the kinds of virus, the percentages of GFP^+^ cells were monitored with FACS before BMT. FACS data showed that the two kinds of virus own similar transduction efficiencies on BM cells (Figure S7A). All the recipients that received BM that was transduced with BCR/ABL1 alone died of B-ALL with a median survival time of 38 days (post BMT); whereas the median survival time for mice that received BCR/ABL1-Rora transduced BM cells was 50 days (Figure 7A). PB leukemia cells (GFP^+^B220^+^) for the recipient mice was monitored on days 15, 18, 22, 25 and 29 post BMT, the percentage and total number of leukemia cells was significantly lower in BCR/ABL1-Rora group recipients (Figure 7B and C). Again, three recipients from each group were sacrificed on day 25, which revealed that the BCR/ABL1-Rora group recipients had a smaller gross splenic appearance with a significantly lower weight (Figure 7D and E) and significantly lower percentage and total number of leukemia cells (Figure 6F and G). The data from the BCR/ABL1-Rora group BM was consistent with splenic data (Figure 7H and I). Taken together, these results suggest that high *Rora* expression impeded BCR/ABL1-induced B-ALL progression *in vivo*.

## Discussion

*Rora* is an important transcription factor that had previously not been studied in the context of B cell development. Herein, *Rora* deficiency was shown to promote the regenerative capacity of B cells, resulting higher percentages in the PB, SPL and BM. *Rora* was also demonstrated to participate in ph^+^ B-ALL pathogenesis using a BCR/ABL1-transduced B-ALL mouse model. *Rora* deficiency resulted in a more aggressive disease progression for B-ALL; whereas Rora over-expression diminished the pathogenicity of BCR/ABL1- induced B-ALL.

Previous studies support the notion that RORA functions as a tumor suppressor in B cell leukemia, since RORA is known to repress WNT/B-catenin signaling^8^, which is essential for BCR/ABL1-transduced leukemia^23^. Moreover, its tumor suppressor activity is supported in a broader sense because in colorectal cancer studies, RORA was observed to inhibit cancer cell growth by increasing TP53 stability, which causes cancer cell apoptosis is response to DNA damage signals^6^. RORA interacts with nucleoside diphosphate kinases, including NM23-1 and NM23-2. NM23-1 is a tumor metastasis suppressor candidate gene^24^,^25^, and NM23-2 plays a role in organogenesis and differentiation^26^,^27^. RORA is also a potential target in breast cancer that negatively modulates angiogenesis^11^. Further mechanistic investigation into the role of these genes and pathways in the context of RORA's involvement with B-ALL may be required. Interesting, here we firstly find that Rora may inhibits Lmo1 transcription (Figure 3A). Previous studies have demonstrated that genetic variants of LMO1 are associated with ALL and it may be a strong candidate for precursor B-cell leukemogenesis^28^. It is worth to investigate the function of LMO1 in B cell development and BCR/ABL1-transduce leukemogenesis and progress in near future.

B cell development is a coordinated process and is controlled by multiple regulatory networks. During this process, various transcription factors are considered to play an essential role with respect to the stage and lineage specific differentiation of B cells. Studies with mice bearing target mutations have demonstrated that a number of transcription factors are crucial in the regulation of normal B lymphocyte differentiation, including Pax5^29^, Ikzf1^30^, Tcf3^31^,^32^, Myb^33^, Spi1^34^, and Ebf1^35^. Disruptions of these transcription factors are associated with abnormal B cell phenotypes that can cause either developmental arrest or the development of B lineage leukemias. To date no evidence is available that supports the notion that Rora directly regulates *Pax5*, *Ikzf1*, *Tcf3*, *Myb* or *Ebf1*, nor have they been reported to regulate *Rora*.

Previous studies have revealed that exogenously expressed RORA and use of agonists (such as melatonin or cholesterol sulfate) is associated with the enhanced transcriptional activity of HIF1A via association with its DNA binding domain^7^. Hypoxia-inducible factors are major transcription factors responding hypoxia^36^ and tend to be highly expressed during B cell differentiation, particularly at the pro-and pre-B stages; whereas they are decreased at the immature B cell stage. This stage-specific expression patten of HIF-1α is required for normal B cell development and Hif-1a deficiency is known to result in reduced BCR repertoire diversity, the developmental arrest of immature B cells and a decreased number of B cells in the PB^37^. However, *Rora* deficiency resulted in an increase of B cell numbers in PB, SPL and BM, therefore it may not impact B cell development through Hif-1a. Clinical data has indicated that higher mRNA level of *Hif-1a* is associated with a higher 5-year event-free survival rate for childhood B-ALL^38^. This matches the observations made herein, where higher expression of RORA impeded B-ALL progression *in vivo*. Ultimately, there appears to be no evidence to support the involvement of Hif-1a with Rora with regards to B cell development or B-ALL pathogenesis. Nevertheless, the models described herein may provide a system to investigate the relationship between Rora and Hif-1a regarding the regulation of B cell development and B-ALL pathogenesis.

In conclusion, ablation of expression *Rora* was shown to promote Ph^+^ B-ALL progression using BCR/ABL1-induced B-ALL mouse models; whereas activation of Rora with a specific agonist (CS) inhibited Ph^+^ B-ALL progression. This study therefore demonstrates the potential applicability of RORA as therapeutic target for Ph^+^ B-ALL therapy, for which a curative therapy has yet to be developed.

## Supplementary Material

Supplementary figures.

Supplementary table 1 (BM Pro-B cell).

Supplementary table 2 (B-ALL B cell).
