# Supplementary material for: The suppressive functions of Rora in B lineage cell proliferation and BCR/ABL1-induced B-ALL pathogenesis
Source: Int J Biol Sci. 2022 Mar 6;18(6):2277–91. doi: 10.7150/ijbs.68939 (PMC8990459; doi:10.7150/ijbs.68939)

Figure S1-1.

***Rora* deficiency increases B cell proliferation.** (**A**) A summary of the *Rora* conditional knockout mouse strategy. (**B**) Southern blot results for the *Rora* alteration, which indicates those with correct recombinant, random insertion and wild type genotyping as indicated. (**C**) Mouse crossing strategy to produce *Rora* deficient HSCs and the genotyping results as indicated.

Figure S1-2.

***Rora* deficiency increases B cell proliferation.** (A)PBMC cells isolated from $Rora^{loxp/loxp}$ and Mx-1-Cre/$Rora^{loxp/loxp}$ mice post Pipc treatment for 4 weeks. The deletion efficiency of Rora was examined using immunofluorescence staining with a Rora antibody, Rora (green), DAPI, and merged. (B and C) The total numbers of B cells ($B220^{+}$) in BM and SPL from $\mathrm{Rora}^{\mathrm{loxp/loxp}}$ and Mx-1-Cre/$Rora^{loxp/loxp}$ mice at four, eight, and 12 weeks post Pipc injection. 3-5 mice were included in each group.

Figure S4.

The activation of Rora with CS inhibits B cell proliferation and differentiation. (**A-C**) total number of mature B cells ($B220^{+}$) in the BM, SPL and PB of 10-week old mice treated with CS for seven days. Each independent experiment was repeated three times. All values were represented using the mean ±SEM, for which the Mann Whitney U test was used to evaluate the significance with a threshold of P=<0.05 (*).

Figure S6.

Gene expression signatures of *Rora* deficiency BCR/ABl1 transduced pro-B cells. (A) Heat maps showing the expression changes of top 98 genes andhierarchical clustering of the genes in *Rora* deficiency BCR/ABL1 transduced pro-B cells from the two biological replicates. Comparison of the global gene transcription profiles of *Rora* deficiency leukemia cells at week four Pipc injection. (B-E) Gene Set Enrichment Analysis showsen richment of gene sets upregulated in *Rora* deficiency leukemia cells. (F) Expression level of genes from selected gene sets were confirmed usingquantitative qPCR. Data are expressed as the means±SD of triplicate experiments performed at one time.

Figure S7.

High expression of Rora prevented BCR/ABL1-transduced B-ALL procession *in vivo*.
Viral transduction efficiencies of BCR/ABL or BCR/ABL1-Rora viral stock on WT BM cells for B-ALL mouse model.

Fig.S1-1

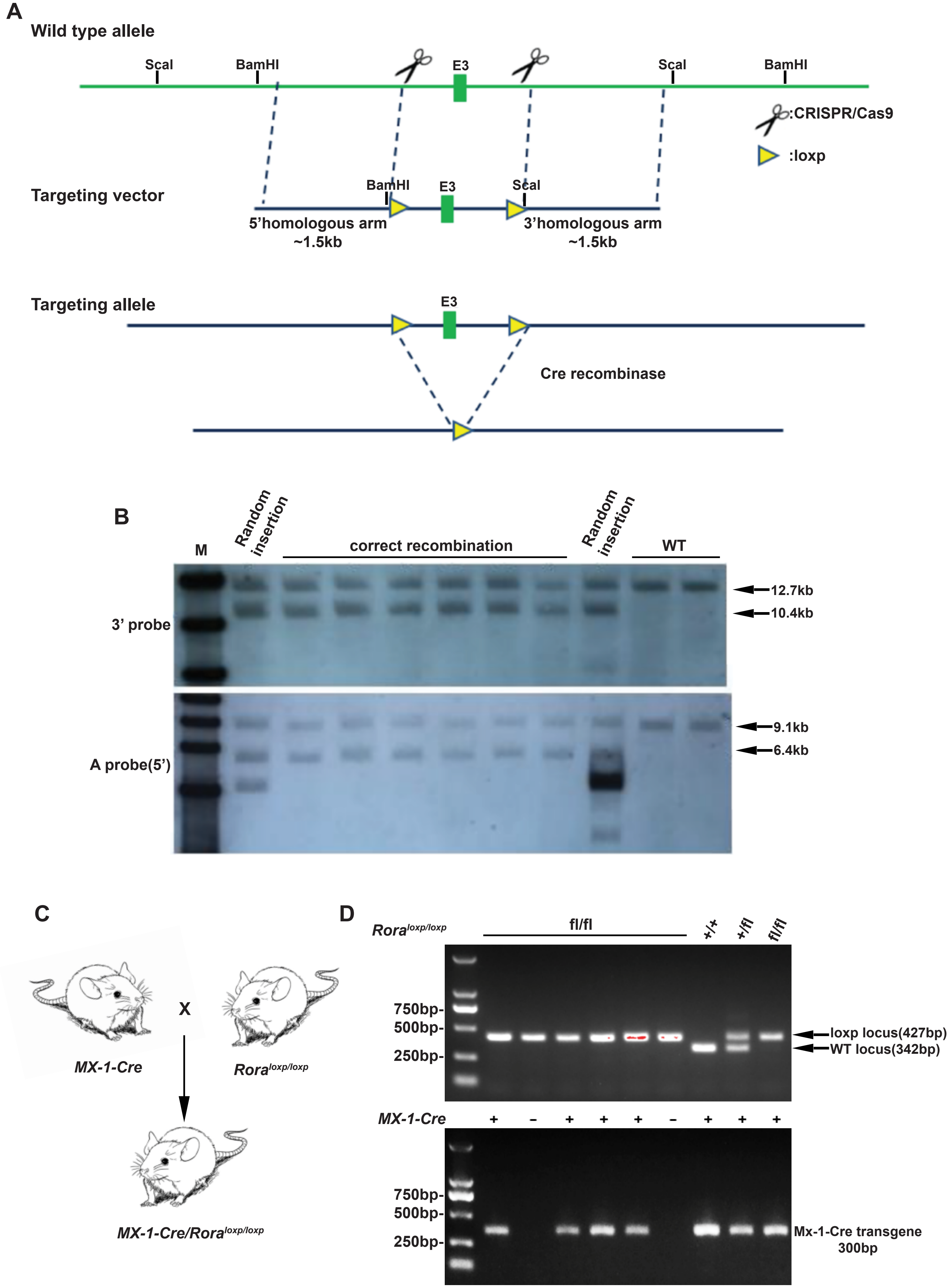

A
Wild type allele
ScaI
BamHI
E3
ScaI
BamHI
:CRISPR/Cas9
:loxp
Targeting vector
BamHI
E3
ScaI
5'homologous arm
~1.5kb
3'homologous arm
~1.5kb
Targeting allele
E3
Cre recombinase
B
M
Random insertion
correct recombination
Random insertion
WT
3' probe
12.7kb
10.4kb
A probe(5')
9.1kb
6.4kb
C
X
MX-1-Cre
Rora loxp/loxp
MX-1-Cre/Rora loxp/loxp
D
Rora loxp/loxp
fl/fl
+/+
+/fl
fl/fl
750bp-
500bp-
250bp-
loxp locus(427bp)
WT locus(342bp)
MX-1-Cre
+ – + + + – + + +
750bp-
500bp-
250bp-
Mx-1-Cre transgene
300bp

Fig.S1-2

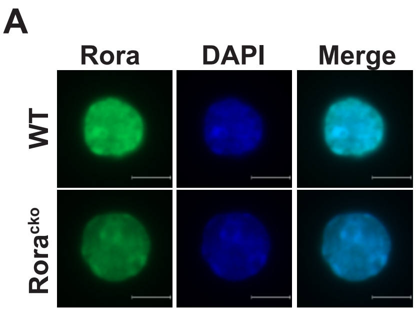
A
Rora
DAPI
Merge
WT
Rora^{cko}

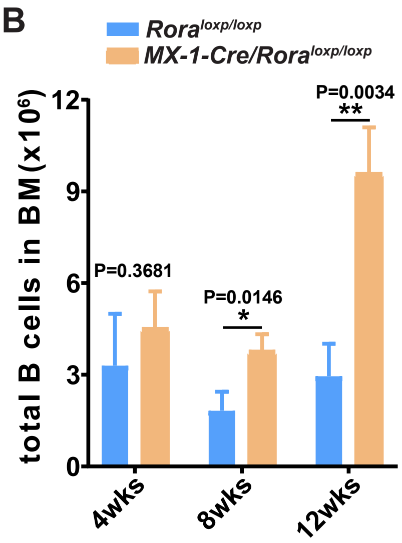
B
Rora$^{loxp/loxp}$
MX-1-Cre/Rora$^{loxp/loxp}$
total B cells in BM(x10^6)
12
9
6
3
0
P=0.3681
P=0.0146
*
P=0.0034
**
4wks
8wks
12wks

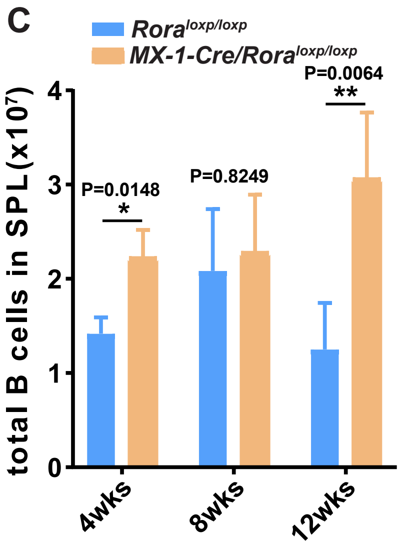
C
Rora$^{loxp/loxp}$
MX-1-Cre/Rora$^{loxp/loxp}$
total B cells in SPL(x10^7)
4
3
2
1
0
P=0.0148
*
P=0.8249
P=0.0064
**
4wks
8wks
12wks

Fig.S4

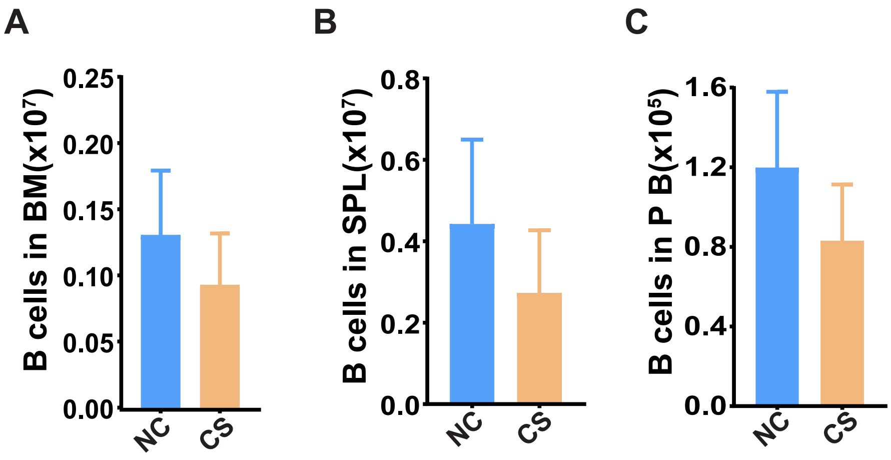

A
B cells in BM(x10^7)
0.25
0.20
0.15
0.10
0.05
0.00
NC
CS
B
B cells in SPL(x10^7)
0.8
0.6
0.4
0.2
0.0
NC
CS
C
B cells in P B(x10^5)
1.6
1.2
0.8
0.4
0.0
NC
CS

Fig.S6

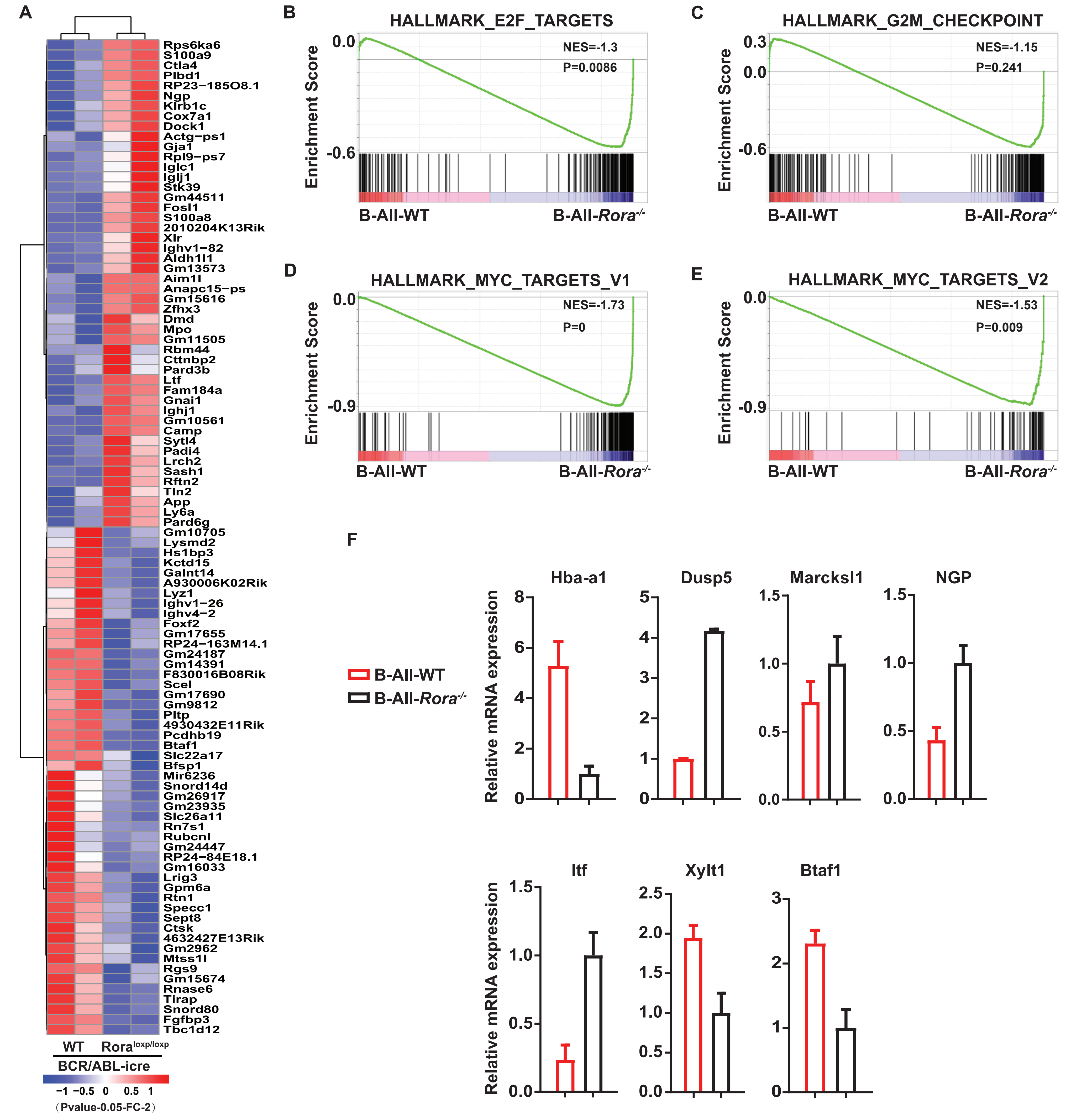

A
Rps6ka6
S100a9
Ctla4
Plbd1
RP23−185O8.1
Ngp
Klrb1c
Cox7a1
Dock1
Actg−ps1
Gja1
Rpl9−ps7
Iglc1
Iglj1
Stk39
Gm44511
Fosl1
S100a8
2010204K13Rik
Xlr
Ighv1−82
Aldh1l1
Gm13573
Aim1l
Anapc15−ps
Gm15616
Zfhx3
Dmd
Mpo
Gm11505
Rbm44
Cttnbp2
Pard3b
Ltf
Fam184a
Gnai1
Ighj1
Gm10561
Camp
Sytl4
Padi4
Lrch2
Sash1
Rftn2
Tln2
App
Ly6a
Pard6g
Gm10705
Lysmd2
Hs1bp3
Kctd15
Galnt14
A930006K02Rik
Lyz1
Ighv1−26
Ighv4−2
Foxf2
Gm17655
RP24−163M14.1
Gm24187
Gm14391
F830016B08Rik
Scel
Gm17690
Gm9812
Pltp
4930432E11Rik
Pcdhb19
Btaf1
Slc22a17
Bfsp1
Mir6236
Snord14d
Gm26917
Gm23935
Slc26a11
Rn7s1
Rubcnl
Gm24447
RP24−84E18.1
Gm16033
Lrig3
Gpm6a
Rtn1
Specc1
Sept8
Ctsk
4632427E13Rik
Gm2962
Mtss1l
Rgs9
Gm15674
Rnase6
Tirap
Snord80
Fgfbp3
Tbc1d12
WT Rora loxp/loxp
BCR/ABL-icre
−1 −0.5 0 0.5 1
（Pvalue-0.05-FC-2）
B
HALLMARK_E2F_TARGETS
Enrichment Score
0.0
-0.6
NES=-1.3
P=0.0086
B-All-WT
B-All-Rora-/-
C
HALLMARK_G2M_CHECKPOINT
Enrichment Score
0.3
0.0
-0.6
NES=-1.15
P=0.241
B-All-WT
B-All-Rora-/-
D
HALLMARK_MYC_TARGETS_V1
Enrichment Score
0.0
-0.9
NES=-1.73
P=0
B-All-WT
B-All-Rora-/-
E
HALLMARK_MYC_TARGETS_V2
Enrichment Score
0.0
-0.9
NES=-1.53
P=0.009
B-All-WT
B-All-Rora-/-
F
B-All-WT
B-All-Rora-/-
Relative mRNA expression
Hba-a1
Dusp5
Marcksl1
NGP
Itf
Xylt1
Btaf1
0 2 4 6 8
0 1 2 3 4 5
0.0 0.5 1.0 1.5
0.0 0.5 1.0 1.5
0.0 0.5 1.0 1.5
0.0 0.5 1.0 1.5 2.0 2.5
0 1 2 3

Fig.S7

A

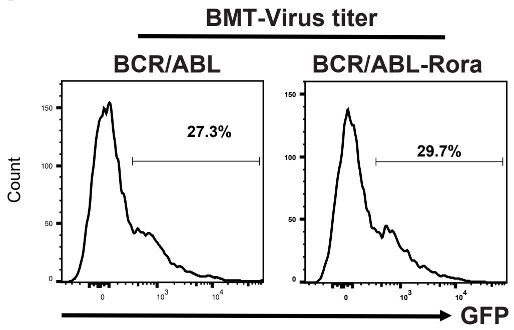

Supplement: Supplementary file 1 — Supplementary figures. [file ijbsv18p2277s1.pdf]
